# Four new coelotine species (Araneae, Agelenidae, Coelotinae) from South China, with the first description of the male of *Coelotes septus* Wang, Yin, Peng & Xie, 1990

**DOI:** 10.3897/zookeys.1029.63060

**Published:** 2021-04-08

**Authors:** Ji-he Liu, Yong-hong Xiao, Meng-zhen Zhang, Xiang Xu, Ke-ke Liu

**Affiliations:** 1 College of Life Science, Hunan Normal University, Changsha 410081, Hunan, China Hunan Normal University Changsha China; 2 College of Life Science, Jinggangshan University, Ji’an 343009, Jiangxi, China Jinggangshan University Ji'an China

**Keywords:** *
Draconarius
*, *
Orumcekia
*, southern China, taxonomy, *
Tonsilla
*, unknown male

## Abstract

Four new species are described from Jinggang Mountain National Nature Reserve, Jiangxi Province of southern China: *Draconarius lingdang***sp. nov.** (♂♀), *D. substrophadatus***sp. nov.** (♀), *Orumcekia cipingensis***sp. nov.** (♀) and *Tonsilla shuikouensis***sp. nov.** (♀). Additionally, *Coelotes septus* Wang, Yin, Peng & Xie, 1990 is redescribed and its male is described for the first time.

## Introduction

The spiders of the family Agelenidae are usually found in a wide range of habitats such as deserts, grasslands, wetlands, and forests and they can live in caves, leaf litter, leaves, humus, bark, brush, streams, forest canopies, tree roots, house, and under rocks. It is a group of entelegyne spiders, currently known by 87 genera and 1342 species (WSC 2020). The species diversity of this family is the highest in Asia (ca. 900 species), from where 67% of total number of agelenid species are reported (Zhu et al. 2017; Li 2020; WSC 2020). More than half (463 species from 35 genera) are described from China (Zhu et al. 2017; Li 2020; WSC 2020). The increased species numbers mainly benefited from active arachnologists’ efforts, such as Shuqiang Li, Zhisheng Zhang, and Xinping Wang (WSC 2017–2020). Among Chinese agelenid species, most (ca. 400 species) are reported from the south provinces of the country (Chen et al. 2015a, b, 2016; Zhang et al. 2016, 2017; Zhao and Li 2016; Zhang and Zhao 2017; Zhu et al. 2017; Li et al. 2018a, b, c, 2019a, b, c, d; Yuan et al. 2019). According to the collecting information recorded on WSC (2020), southern China has both the richest species and generic diversities (Zhu et al. 2017); however, there are still many species (more than 200 species) known by one sex only.

Many details of many species in this family were not known until the work by Zhu et al. (2017). At present, many diverse groups of genera were described and reported from south China, such as *Ageleradix* Xu & Li, 2007, *Bifidocoelotes* Wang, 2002, *Flexicoelotes* Chen, Li & Zhao, 2015, *Guilotes* Zhao & Li, 2018, *Huangyuania* Song & Li, 1990, *Leptocoelotes* Wang, 2002, *Longicoelotes* Wang, 2002, *Nuconarius* Zhao & Li, 2018, *Papiliocoelotes* Zhao & Li, 2016, *Robusticoelotes* Wang, 2002, *Sinocoelotes* Zhao & Li, 2016, *Sinodraconarius* Zhao & Li, 2018, *Tonsilla* Wang & Yin, 1992, *Troglocoelotes* Zhao & Li, 2019, and *Vappolotes* Zhao & Li, 2019.

The Jinggang Mountain National Nature Reserve is located in the middle section of Luoxiao Mountains in southern China. While studying its funnel-web spider fauna, four new species and the previously unknown male of *Coelotes septus* Wang, Yin, Peng & Xie, 1990 were found and are described herein.

## Materials and methods

More than 2600 specimens, belonging to 22 species from 12 genera, were collected using sieving and hand sampling. Specimens were examined using a Zeiss Stereo Discovery V12 stereomicroscope with a Zeiss AxioCam HRc. Both the male palps and female copulatory organs were dissected and examined in 75–80% ethanol. The vulvae were cleaned in pancreatin. All the specimens were photographed with a Zeiss Axio Scope A1 compound microscope with a KUY NICE CCD.

All measurements were made by using the software ImageView CM2000 and are given in millimeters. Leg measurements are given as total length (femur, patella, tibia, metatarsus, tarsus). All the specimens are deposited in the Animal Specimen Museum, College of Life Science, Jinggangshan University (**ASM-JGSU**).

Terminology of the male and female copulatory organs follows Wang (2002), Wang (2003), and Wang et al. (2010). The abbreviations used in the text and figures are:

**Eyes**

**ALE** anterior lateral eye;

**AME** anterior median eye;

**PLE** posterior lateral eye;

**PME** posterior median eye.

**Male palp**

**CF** cymbial furrow;

**Con** conductor;

**DAC** dorsal apophysis of conductor;

**Em** embolus;

**MA** median apophysis;

**PA** patellar apophysis;

**RTA** retrolateral tibial apophysis;

**TS** tooth-like sclerite;

**VTA** ventrolateral tibial apophysis.

**Epygine**

**At** atrium;

**CD** copulatory duct;

**CO** copulatory opening;

**EH** epigynal hood;

**ET** epigynal teeth;

**FD** fertilization duct;

**GT** glandular tubes;

**SH** spermathecal head;

**Spe** spermatheca.

## Taxonomy

**Family Agelenidae C.L. Koch, 1837**

### Genus *Coelotes* Blackwall, 1841

#### Coelotes septus

Taxon classificationAnimaliaAraneaeAgelenidae

Wang, Yin, Peng & Xie, 1990

AAB0AAAB-5041-5197-9652-0790078F0915

Figures 1 , 2

Coelotes septus Wang et al. 1990: 224, figs 108–109 (♀); Song et al. 1999: fig. 224P–Q (♀); Wang and Jäger 2007: 34, figs 44–47 (♀); Yin et al. 2012: 996, fig. 510a, b (♀); Zhu et al. 2017: 188, fig. 96A, B (♀).

##### Material examined.

• 1 ♀, China, Jiangxi Prov., Ji’an City, Jinggangshan County Level City, Ciping Town, Dajing Village, Shiliao Cave, 26°34'12.89"N, 114°07'41.87"E, 950 m, 2 Oct. 2018, Ke-ke Liu et al. leg.; • 4 ♀, 1 ♂, same locality, Dajing Village, Jingzhu Mountain, 26°30'10.8"N, 114°5'16.8"E, 1085 m, 20 Dec. 2015, Ke-ke Liu et al. leg.; • 1 ♀, same locality, Lingxiufeng Scenic Spot, 26°34'12"N, 114°7'19.2"E, 947 m, 25 Aug. 2015, Ke-ke Liu et al. leg.; • 1 ♀, same locality, Xiajing Village, 26°34'12"N, 114°7'19.2"E, 927 m, 26 Aug. 2015, Zhi-wu Chen et al. leg.

##### Diagnosis.

**Figure 1.**
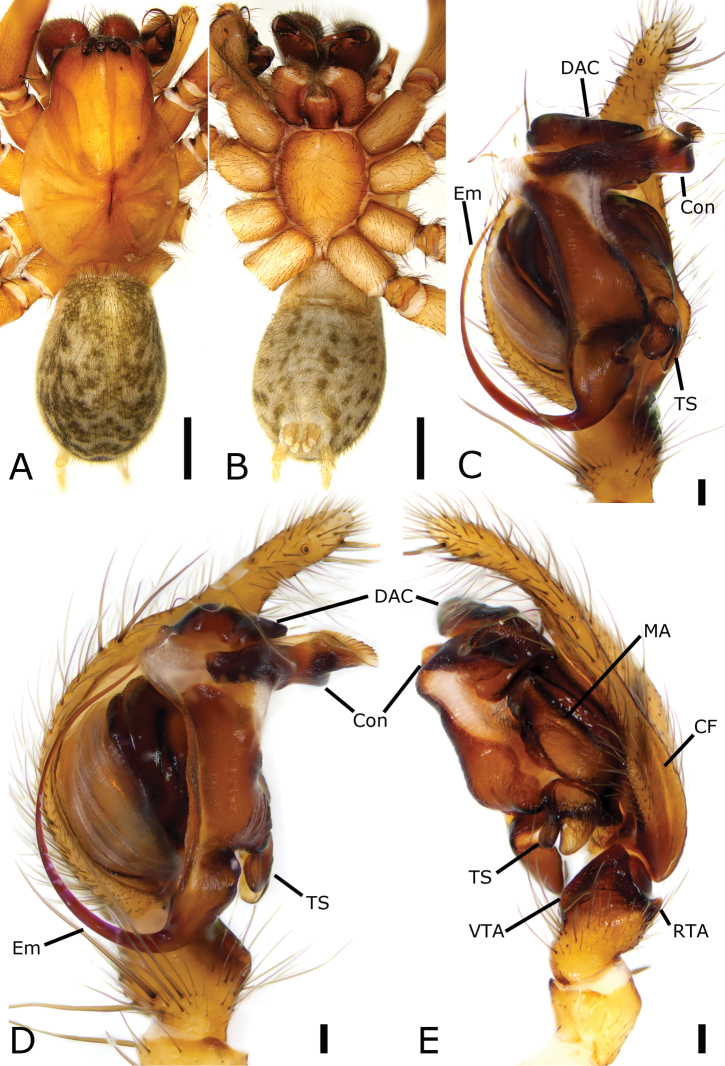
*Coelotes septus*Wang et al., 1990, male **A** habitus, dorsal view **B** same, ventral view **C** palp, ventral view **D** same, prolateral view **E** same, retrolateral view. Scale bars: 1 mm (**A, B**); 0.1 mm (**C–E**). Abbreviations: CF – cymbial furrow, Con – conductor, DAC – dorsal apophysis of conductor, Em – embolus, MA – median apophysis, RTA – retrolateral tibial apophysis, TS – tooth-like sclerite, VTA – ventrolateral tibial apophysis.

**Figure 2.**
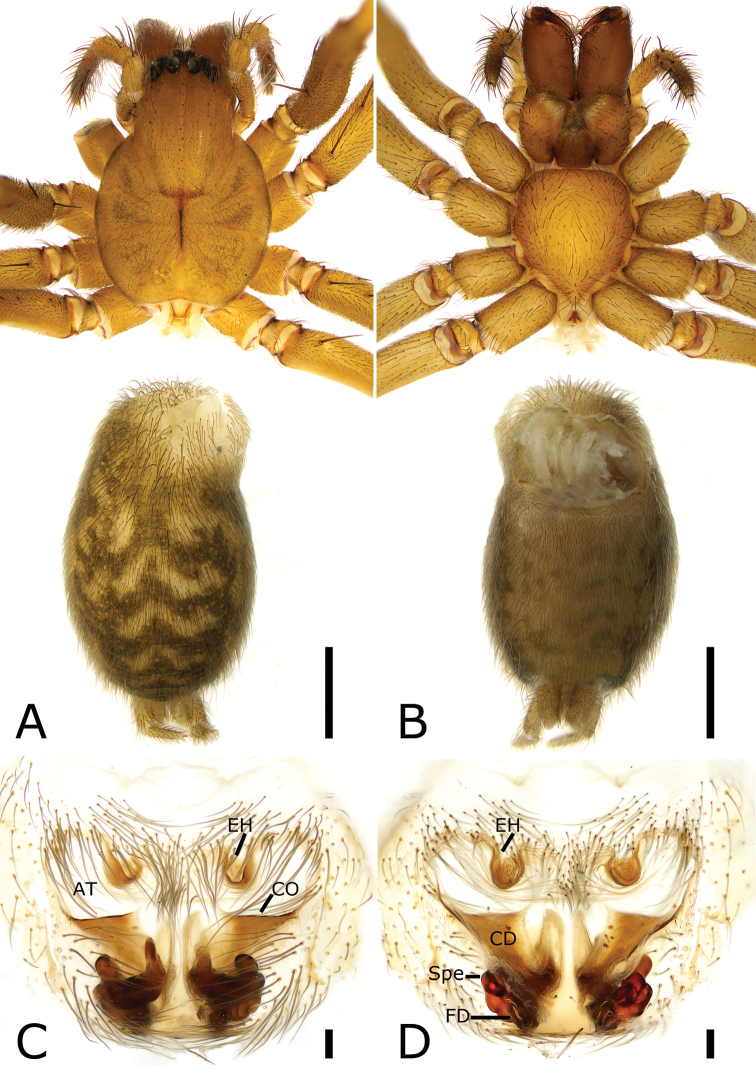
*Coelotes septus*Wang et al., 1990, female **A** habitus, dorsal view **B** same, ventral view **C** epigyne, ventral view **D** vulva, dorsal view. Scale bars: 1 mm (**A, B**); 0.1 mm (**C, D**). Abbreviations: At – atrium, CD – copulatory duct, CO – copulatory opening, EH – epigynal hood, FD – fertilization duct, Spe – spermatheca.

The male resembles *Coelotes conversus* Xu & Li, 2006 in having a long, broad, curved conductor and the small triangular retrolateral apophysis, but can be easily separated by the absence of a patellar apophysis (vs. long patellar apophysis in *C. conversus*), the indistinct median apophysis (vs. spoon-like in *C. conversus*), and a tooth-like sclerite (vs. absent in *C. conversus*) (Fig. 1C–E) on the male palp. The female of this species is easily recognized by the transversal slit-shaped copulatory openings (vs. arc-shaped, longitudinal, or other), and the touching triangular copulatory ducts (vs. tube-shaped, sac-shaped, ellipsoidal, and other) (Fig. 2C, D).

##### Description.

**Male. *Habitus*** as in Fig. 1A, B. Total length 7.12. Carapace 3.66 long, 2.65 wide. Eyes sizes and interdistances: AME 0.09; ALE 0.14; PME 0.14; PLE 0.18; AME–AME 0.06; AME–ALE 0.06; PME–PME 0.08; ALE–ALE 0.34; PME–PLE 0.13; PLE–PLE 0.59; ALE–PLE 0.06; AME–PME 0.06; AME–PLE 0.22. MOA: 0.62 long; 0.56 anterior width, 0.62 posterior width. Chelicerae with three promarginal teeth (median largest) and two retromarginal teeth (distal larger). Leg measurements: I 8.88 (2.22, 0.6, 2.29, 2.27, 1.50); II 8.14 (2.26, 0.52, 1.92, 2.02, 1.42); III broken; IV 10.34 (2.72, 0.84, 1.98, 3.43, 1.37). Abdomen 3.23 long, 2.29 wide.

***Coloration*.** Carapace yellow-brown. Chelicerae red-brown. Endites, labium, and sternum yellow-brown. Legs yellow-brown. Abdomen dark brown, dorsally with five pale chevron stripes on sub-medial part.

***Palp*** (Fig. 1C–E). Femur and patella without apophysis. Tibia with wide ventrolateral apophysis and small retrolateral apophysis. VTA extending beyond half of tibia, strongly sclerotized; RTA small, < 1/5 × length of ventrolateral apophysis, apex pointing to the base of cymbium. Cymbial furrow slightly shorter than half of the cymbial length in retrolateral view. Median apophysis indistinct, slightly protruding. Sclerite, tooth-like in retrolateral view, located near the base of embolus; conductor, extending transversally, apical part hook-shaped and pointing to distal part of cymbium, with many ridges; basal part with a long, curved, sclerotized dorsal apophysis, less than the length of transversal conductor; embolus whip-shaped, originating at the 6 o’clock position, coiled around the margin of cymbium and posteriorly curved and embedded in the furrow of conductor.

**Female. *Habitus*** as in Fig. 2A, B. Total length 5.57. Carapace 2.68 long, 1.93 wide. Eye sizes and interdistances: AME 0.12; ALE 0.14; PME 0.14; PLE 0.15; AME–AME 0.12; AME–ALE 0.06; PME–PME 0.08; ALE–ALE 0.36; PME–PLE 0.07; PLE–PLE 0.50; ALE–PLE 0.06; AME–PME 0.07; AME–PLE 0.19. MOA: 0.21 long; 0.28 anterior width, 0.36 posterior width. Chelicerae with three promarginal teeth (proximal smallest, median largest) and five retromarginal teeth (proximal smallest, sub-distal largest). Leg measurements: I 9.05 (2.45, 0.63, 2.39, 2.08, 1.5); II 8.46 (2.29, 0.74, 2.01, 1.88, 1.54); III 6.97 (2.11, 0.46, 1.79, 1.66, 0.95); IV 8.25 (2.23, 0.54, 2.25, 1.71, 1.52). Pedicel 1.17. Abdomen 3.02 long, 2.03 wide.

***Epigyne*** (Fig. 2C, D). Atrium broad, transversal, anteriorly located. Epigynal hoods concave, located at anterior margin, separated by > 2 × length. Copulatory openings located at postero-lateral of the atrium, transversal, slit-like. Epigynal tooth absent. Copulatory ducts triangular, broad, covering anterior part of spermathecae, extending from copulatory openings to sub-posterior part of spermathecae. Spermathecae sac-shaped, including a few thin tubes, anterior part slightly separated, posterior part separated by less than their maximum widths. Fertilization ducts located at the posterior part of the spermathecae, slightly curved forward laterally.

##### Comments.

This species was described by Yin et al. (1990) based on five female specimens, collected from Yanling County, Hunan Province and was not collected after its original description. Xin-ping Wang (Zhu et al. 2017) re-examined the holotype, providing more detailed characters that make this species more easily identified. *Coelotes septus* does not belong to the two groups recognized from China and defined by Wang (2002), namely the *Coelotes atropos* group and the *Coelotes pseudoterrestri* group. It is also different from other *Coelotes* species in the patellar apophysis and tooth-like sclerite of male palps, epigynal teeth, and transverse slit-like copulatory openings of the female epigyne. Hence, it is possible that it is not a true *Coelotes* species. However, it was placed in the genus *Coelotes* in its initial description; therefore, it is provisionally retained in *Coelotes*.

##### Distribution.

Known only from Jiangxi and Hunan Provinces, China (Fig. 8).

### Genus *Draconarius* Ovtchinnikov, 1999

#### Draconarius lingdang

Taxon classificationAnimaliaAraneaeAgelenidae

K. Liu, J. Liu & X. Xu sp. nov.

9BF98133-1B4C-575C-AF9D-AA4D1012EA73

http://zoobank.org/65A5EA62-AE82-45A7-A35E-A398E4074579

Figures 3 , 4

##### Material examined.

***Holotype*** ♂, China, Jiangxi Prov., Ji’an City, Jinggangshan County Level City, Ciping Town, Xiajing Village, Shuikou Scenic Spot, 26°33'04.83"N, 114°27'42.83"E, 898 m, 1 Dec. 2013, Ke-ke Liu et al. leg. ***Paratype*** 1 ♀, the same data as holotype.

##### Etymology.

The name comes from the Chinese word *lingdang*, meaning bell, referring to the shape of the spermathecae as seen through the ventral cuticle; noun in apposition.

##### Diagnosis.

**Figure 3.**
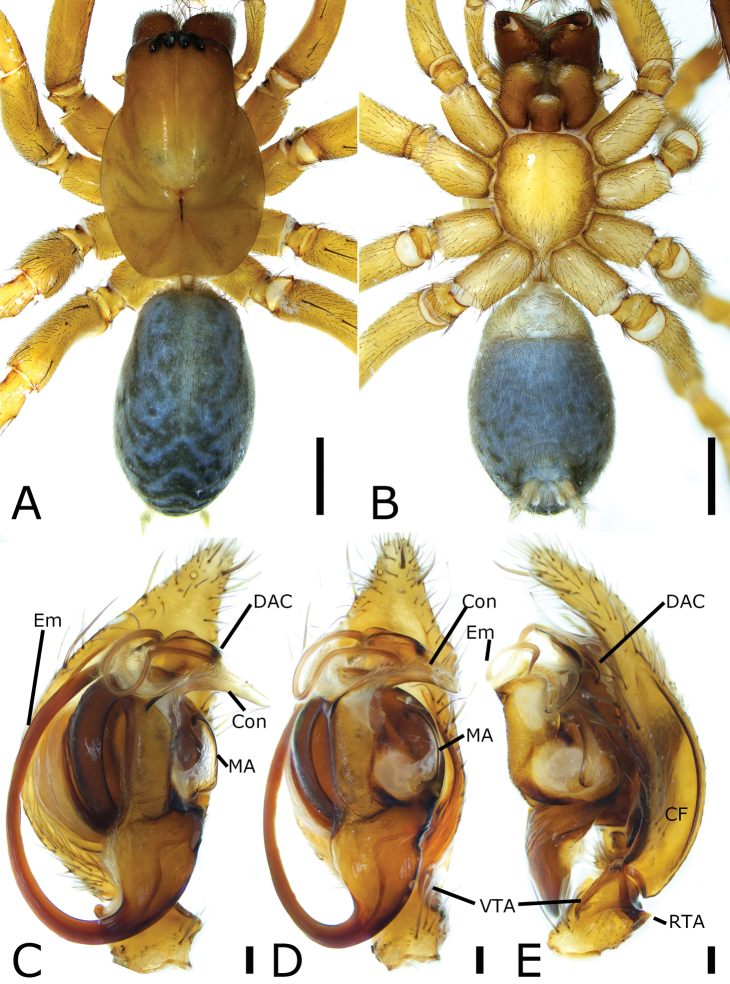
*Draconarius lingdang* sp. nov., male holotype **A** habitus, dorsal view **B** same, ventral view **C** palp, prolateral view **D** same, ventral view **E** same, retrolateral view. Scale bars: 1 mm (**A, B**); 0.1 mm (**C–E**). Abbreviations: CF – cymbial furrow, Con – conductor, DAC – dorsal apophysis of conductor, Em – embolus, MA – median apophysis, RTA – retrolateral tibial apophysis, VTA – ventrolateral tibial apophysis.

**Figure 4.**
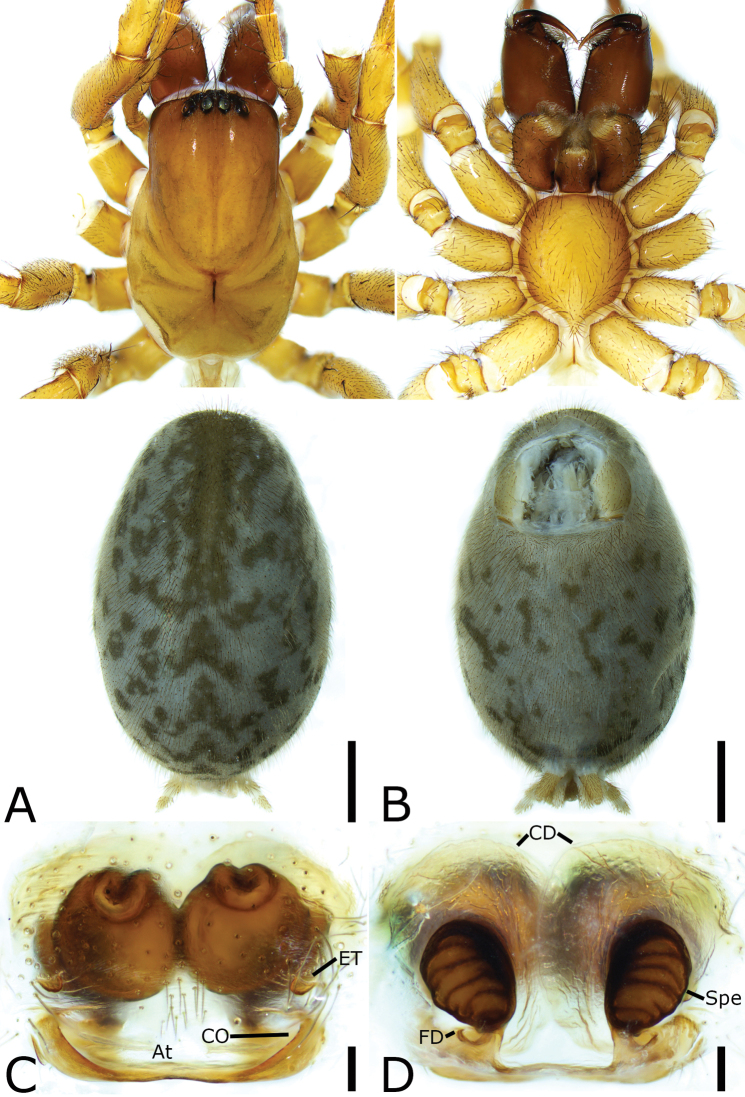
*Draconarius lingdang* sp. nov., female paratype **A** habitus, dorsal view **B** same, ventral view **C** epigyne, ventral view **D** vulva, dorsal view. Scale bars: 1 mm (**A, B**); 0.1 mm (**C, D**). Abbreviations: At – atrium, CD – copulatory duct, CO – copulatory opening, ET – epigynal teeth, FD – fertilization duct, Spe – spermatheca.

The male of this species is similar to that of *D. potanini* (Schenkel, 1963) in having the whip-like embolus with two turns in the anterior part and the absence of a patellar apophysis, but differs by the broad distal groove of conductor (vs. narrow in *D. potanini*) and the triangular and sharp retrolateral tibial apophysis (vs. broad and blunt in *D. potanini*) (Fig. 3C–E). The male of this species also resembles those of *D. peregrinus* Xie & Chen, 2011 by its long conductor with a triangular distal groove and the absence of a patellar apophysis, but can be separated from it by the embolus extending along the inner margin of cymbium and the distal part with two turns (vs. extending along ectal margin of cymbium and without turn in *D. peregrinus*) (Fig. 3C–E). The female of this species resembles those of *D. peregrinus* in the transparent copulatory ducts wrapping around spermathecae, but differs by the short pocket-shaped epigynal teeth (vs. horn-like in *D. peregrinus*), and the oval spermathecae (vs. elongated ellipsoid in *D. peregrinus*) (Fig. 4C, D).

##### Description.

**Male. *Habitus*** as in Fig. 3A, B. Total length 6.63. Carapace 3.16 long, 2.10 wide. Eye sizes and interdistances: AME 0.09; ALE 0.14; PME 0.14; PLE 0.18; AME–AME 0.06; AME–ALE 0.07; PME–PME 0.08; ALE–ALE 0.34; PME–PLE 0.10; PLE–PLE 0.45; ALE–PLE 0.03; AME–PME 0.05; AME–PLE 0.21. MOA: 0.33 long; 0.22 anterior width, 0.35 posterior width. Chelicerae with three promarginal teeth (median largest) and two retromarginal teeth (distal larger). Leg measurements: I 7.30 (1.98, 0.88, 1.94, 1.73, 0.77); II 5.55 (1.14, 0.93, 1.4, 1.3, 0.78); III 5.92 (1.01, 0.73, 1.53, 1.46, 1.19); IV 7.24 (1.52, 0.95, 2.33, 1.55, 0.89). Abdomen 2.96 long, 1.90 wide.

***Coloration*.** Carapace yellow-brown, posteriorly with dark radial stripes. Chelicerae red-brown. Endites and labium dark yellow-brown. Sternum yellow-brown. Legs yellow. Abdomen dark brown, dorsally with five pale chevron stripes on sub-medial part.

***Palp*** (Fig. 3C–E). Femur and patella without apophysis. Tibia with triangular ventrolateral and short retrolateral apophyses. VTA extending beyond half of tibia, strongly sclerotized. RTA small, < 1/3 × length of ventrolateral one. Cymbial furrow slightly < 1/2 cymbial length in retrolateral view. Median apophysis spoon-like in retrolateral view, located between the base of embolus and conductor; conductor extending transversally, apical part curved forward towards median apophysis in retrolateral view, with a curved furrow; basal part with a strong, sclerotized dorsal apophysis, shorter than the length of transverse conductor; embolus broad, originates at the 6 o’clock position, coiled around the margin of cymbium and posteriorly convoluted and embedded in the furrow of conductor.

**Female (Paratype). *Habitus*** as in Fig. 4A, B. Total length 8.65. Carapace 3.42 long, 2.12 wide. Eye sizes and interdistances: AME 0.13; ALE 0.16; PME 0.14; PLE 0.15; AME–AME 0.07; AME–ALE 0.19; PME–PME 0.08; ALE–ALE 0.36; PME–PLE 0.10; PLE–PLE 0.55; ALE–PLE 0.06; AME–PME 0.07; AME–PLE 0.19. MOA: 0.29 long; 0.25 anterior width, 0.36 posterior width. Chelicerae with three promarginal teeth (proximal smallest, median largest) and two retromarginal teeth (proximal larger). Leg measurements: I 6.72 (1.3, 0.96, 1.91, 1.6, 0.95); II 6.2 (1.49, 0.83, 1.59, 1.43, 0.86); III 5.68 (1.14, 0.86, 1.27, 1.56, 0.85); IV 7.64 (1.26, 0.98, 1.92, 2.44, 1.04). Pedicel 2.84. Abdomen 4.63 long, 2.95 wide.

***Epigyne*** (Fig. 4C, D). Atrium, broad, > 3 × wider than its length, labium-shaped, anterior margin near the apex of teeth, posteromedial part relatively straight. Copulatory openings located at postero-lateral of the atrium. Epigynal teeth flat, very short, pocket-shaped, separated by less than the maximum length of atrium. Copulatory ducts broad, transparent, originating postero-laterally, extending antero-medially around spermathecae, then back, connecting with anterior part of spermathecae. Spermathecae sac-shaped, the distance between them more than their widths. Fertilization ducts located at the posterior part of the spermathecae, curved postero-laterally.

##### Comments.

Patellar apophysis is absent in the male palp of this species, as well as in *Draconarius aspinatus* (Wang, Yin, Peng & Xie, 1990), *D. peregrinus* Xie & Chen, 2011, *D. potanini*, *D. rufulus* (Wang, Yin, Peng & Xie, 1990), *D. subabsentis* Xu & Li, 2008 and *D. tiantangensis* Xie & Chen, 2011, all recorded from China. It seems that they are different from most *Draconarius* which have a clear PA in male palp. All of them are likely to belong to the same species group.

##### Distribution.

Known only from the type locality in Jiangxi Province, China (Fig. 8).

#### Draconarius substrophadatus

Taxon classificationAnimaliaAraneaeAgelenidae

K. Liu, J. Liu & X. Xu sp. nov.

FD87DF7C-8CC6-5ED4-9F9E-8194A7E56C39

http://zoobank.org/C6F360E9-8144-4878-9C84-502ADE78A6D5

Figure 5

##### Material examined.

***Holotype*** ♀, China, Jiangxi Prov., Ji’an City, Jinggangshan County Level City, Ciping Town, Dajing Village, Dajing Forest Farm, 26°34'12"N, 114°7'19.2"E, 956 m, 27 Aug. 2015, Zhi-wu Chen et al. leg.

##### Etymology.

The name refers to its similarity to *D. strophadatus* (Zhu & Wang, 1991).

##### Diagnosis.

**Figure 5.**
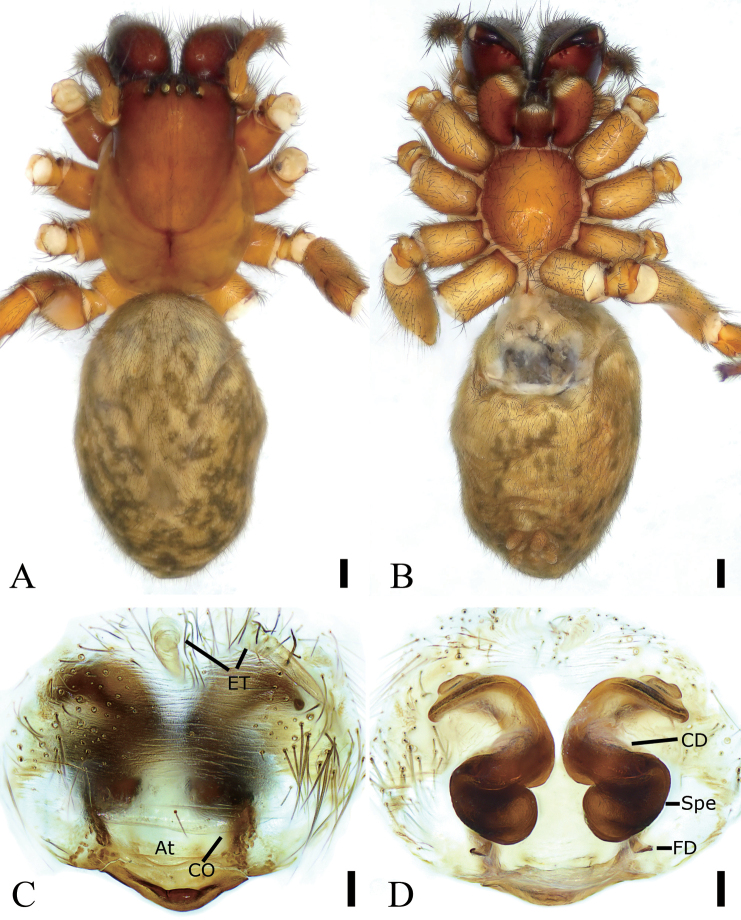
*Draconarius substrophadatus* sp. nov., female holotype **A** habitus, dorsal view **B** same, ventral view **C** epigyne, ventral view **D** vulva, dorsal view. Scale bars: 1 mm (**A, B**); 0.1 mm (**C, D**). Abbreviations: At – atrium, CD – copulatory duct, CO – copulatory opening, ET – epigynal teeth, FD – fertilization duct, Spe – spermatheca.

The female of this species is similar to that of *D. strophadatus* in having the labium-like atrium, the long horn-shaped epigynal teeth and the waved copulatory ducts, but differs by the epigynal teeth separated by 1/2 their length (vs. 1/4 in *D. strophadatus*) and the copulatory ducts from sub-anterior part of vulvae extending to median part forming a C-shaped turn (vs. from anterior part of vulvae extending to median part forming a S-shaped turn in *D. strophadatus*) (Fig. 5C, D).

##### Description.

***Habitus*** as in Fig. 5A, B. Total length 8.4. Carapace 3.74 long, 2.64 wide. Eye sizes and interdistances: AME 0.08; ALE 0.21; PME 0.14; PLE 0.19; AME–AME 0.06; AME–ALE 0.08; PME–PME 0.14; ALE–ALE 0.40; PME–PLE 0.13; PLE–PLE 0.59; ALE–PLE 0.13; AME–PME 0.15; AME–PLE 0.29. MOA: 0.41 long; 0.24 anterior width, 0.30 posterior width. Chelicerae with a large basial tubercle, three promarginal teeth (median largest) and two retromarginal teeth (proximal larger). Leg measurements: I 10.16 (2.72, 1.36, 2.2, 2.75, 1.13); II 8.59 (2.26, 1.05, 1.5, 2.45, 1.33); III 8.18 (2.31, 1, 1.81, 1.89, 1.17); IV 9.32 (2.5, 1.05, 2.28, 2.14, 1.35). Abdomen 4.48 long, 2.08 wide.

***Coloration*.** Carapace yellow-brown, posteriorly with dark, narrow, radial stripes. Chelicerae dark yellow-brown. Endites, labium, and sternum dark yellow-brown. Legs yellow-brown. Abdomen brown, dorsally with six pale chevron stripes on the sub-medial part.

***Epigyne*** (Fig. 5C, D). Atrium, labium-like, arising from posterior. Epigynal teeth long, horn-like, located at anteromedian of epigynum. Copulatory openings located at sub-posterior part of the atrium, covered by a transverse plate. Copulatory ducts S-shaped, extending from sub-posterior to antero-lateral part of vulva, then back, connecting with anterolateral part of spermathecae. Spermathecae ampullate, separated by less than their lengths. Fertilization duct short, located posteriorly on spermathecae, curved posteriorly.

**Male.** Unknown.

##### Comments.

Unfortunately, only one specimen of *Draconarius substrophadatus* sp. nov. was found, as well as of *D. strophadatus*. They share the similar characters of long epigynal teeth, the labium-like atrium located posteriorly, and the waved copulatory ducts, and the new species is tentatively placed in the genus *Draconarius*. Hopefully, finding the males of these two species in the future will reveal their generic placement.

##### Distribution.

Known only from the type locality in Jiangxi Province, China (Fig. 8).

### Genus *Orumcekia* Koçak & Kemal, 2008

There are seven species currently assigned to this genus and they are all distributed in Asia, including China, Thailand, and Vietnam. They are all reported from China except *O. lanna* (Dankittipakul, Sonthichai & Wang, 2006) and characterised by the male palp with two patellar apophyses, a single tibial apophysis, and the female epigyne without epigynal teeth and with a transversal enlarging atrial ridge. It is worth mentioning that most of them are known by a single female only: *O. jianhuii* (Tang & Yin, 2002; Hunan), *O. lanna* (Thailand), *O. pseudogemata* (Xu & Li, 2007; Sichuan), and *O. subsigillata* (Wang, 2003; Zhejiang). Thus, they still need to be supplemented by the other sex in future collections.

#### Orumcekia cipingensis

Taxon classificationAnimaliaAraneaeAgelenidae

K. Liu, J. Liu & X. Xu sp. nov.

9E565FD5-D05A-5E3D-A63E-F11AFC26BEA4

http://zoobank.org/C6FCE1D4-3FF6-4C32-940C-9B343F3D99BF

Figure 6

##### Material examined.

***Holotype*** ♀, China, Jiangxi Prov., Ji’an City, Jinggangshan County Level City, Ciping Town, near Youth Quality Training Camp, 26°35'10.87"N, 114°09'42.52"E, 885 m, 27 Sep. 2018, Ke-ke Liu leg.

##### Etymology.

The name refers to the type locality, Ciping Town; adjective.

##### Diagnosis.

**Figure 6.**
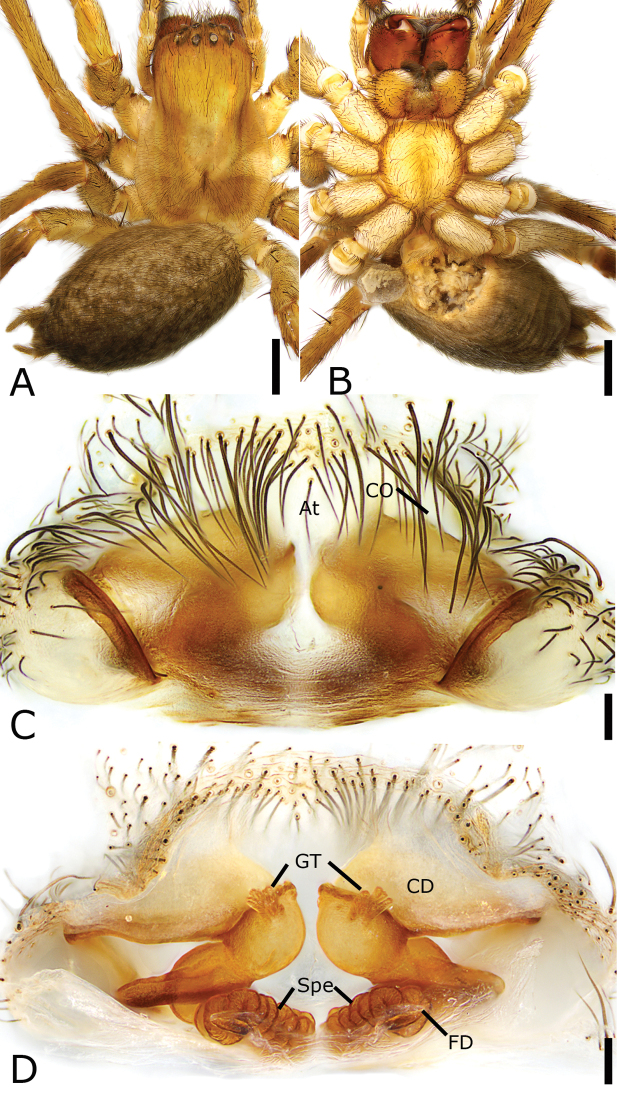
*Orumcekia cipingensis* sp. nov., female holotype **A** habitus, dorsal view **B** same, ventral view **C** epigyne, ventral view **D** vulva, dorsal view. Scale bars: 1 mm (**A, B**); 0.1 mm (**C, D**). Abbreviations: At – atrium, CD – copulatory duct, CO – copulatory opening, FD – fertilization duct, GT – glandular tubes, Spe – spermatheca.

The female of this species is similar to that of *O. gemata* (Wang, 1994), the type species of the genus, in having the broad bugle-shaped copulatory ducts and touching sac-shaped posterior spermathecae, but differs by the longer copulatory duct with a spiral tube (vs. absent in *O. gemata*) and the sac-shaped spermathecae with the anterior peanut-shaped parts slightly separated from each other (vs. Y-shaped parts touching in *O. gemata*) (Fig. 6C, D).

##### Description.

**Female. *Habitus*** as in Fig. 6A, B. Total length 7.71. Carapace 3.34 long, 2.40 wide. Eye sizes and interdistances: AME 0.14; ALE 0.21; PME 0.16; PLE 0.16; AME–AME 0.19; AME–ALE 0.10; PME–PME 0.19; ALE–ALE 0.61; PME–PLE 0.27; PLE–PLE 0.96; ALE–PLE 0.09; AME–PME 0.20; AME–PLE 0.29. MOA: 0.46 long; 0.44 anterior width, 0.48 posterior width. Chelicerae with a large basial tubercle, three promarginal teeth (median largest) and five retromarginal teeth (distal largest). Leg measurements: I 9.74 (2.61, 1.21, 2.48, 2.33, 1.11); II 9.09 (2.67, 0.95, 2.12, 2.15, 1.20); III 7.5 (2.07, 0.94, 1.49, 2.03, 0.97); IV 9.88 (2.5, 1.18, 2.38, 2.47, 1.35). Abdomen 3.78 long, 2.09 wide.

***Coloration*.** Carapace yellow-brown, posteriorly with radial stripes. Chelicerae red-brown. Endites, labium, and sternum yellow-brown. Legs yellow-brown. Abdomen brown, dorsally with six pale chevron stripes on sub-medial part.

***Epigyne*** (Fig. 6C, D). Atrium broad, subfan-shaped, extending from anterior to posterior. Copulatory openings located at mediolateral part of the atrium. Copulatory ducts, anterior part bugle-shaped, posterior part connecting with a spiral tub, longer than spermathecae. Glandular tubes clustered, located at anterior part of spermathecae. Spermathecae in two pairs, anterior spermathecae peanut-shaped, slightly separated, sloping postero-laterally; posterior spermathecae sac-shaped, distal parts touching. Fertilization duct short, located medially on spermathecae.

##### Comments.

A cluster of blind tubes located on the anterior part of the spermathecae of this species is unclear to us; we called them glandular tubes. They are probably homologous with spermathecal heads also originated from spermathecae.

##### Distribution.

Known only from the type locality in Jiangxi Province, China (Fig. 8).

#### Tonsilla shuikouensis

Taxon classificationAnimaliaAraneaeAgelenidae

K. Liu, J. Liu & X. Xu sp. nov.

2967B22C-C48B-576C-896D-31CE571E15E9

http://zoobank.org/243D9BAB-2626-4198-BAFD-46B49FFB2710

Figure 7

##### Material examined.

***Holotype*** ♀, China, Jiangxi Prov., Ji’an City, Jinggangshan County Level City, Ciping Town, Xiajing Village, Shuikou Scenic Spot, 26°33'04.83"N, 114°27'42.83"E, 898 m, 7 Dec. 2013, Ke-ke Liu et al. leg.

##### Etymology.

The name refers to the type locality, Shuikou Scenic Spot; adjective.

##### Diagnosis.

**Figure 7.**
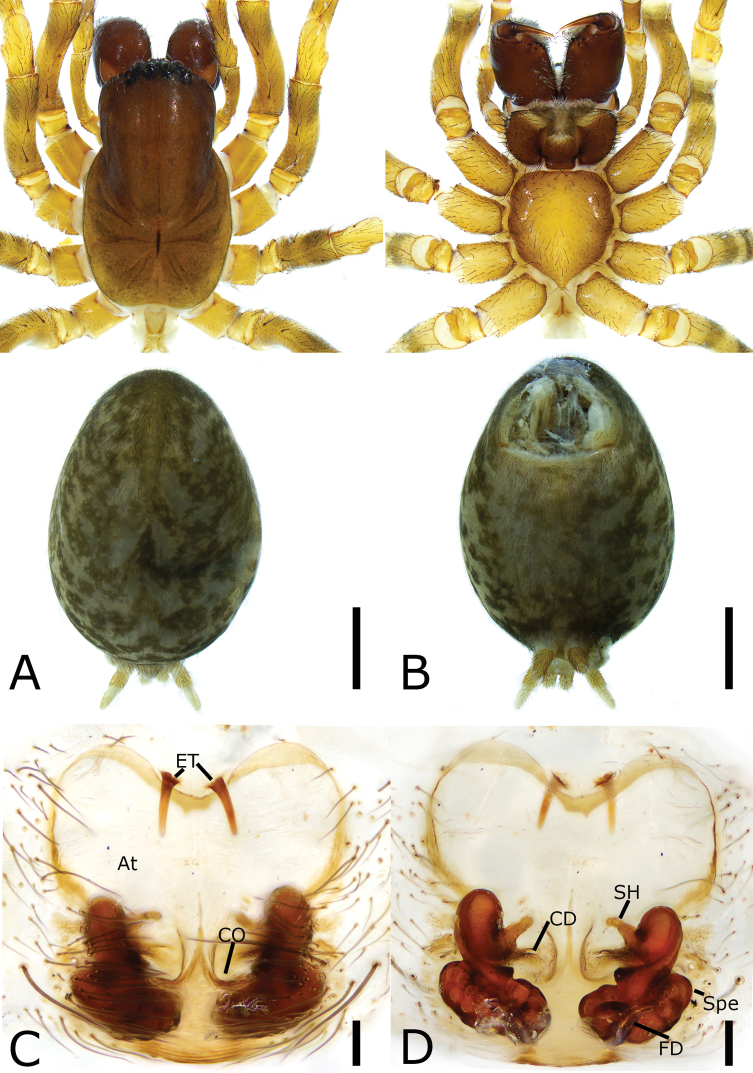
*Tonsilla shuikouensis* sp. nov., female holotype **A** habitus, dorsal view **B** same, ventral view **C** epigyne, ventral view **D** vulva, dorsal view. Scale bars: 1 mm (**A, B**); 0.1 mm (**C, D**). Abbreviations: At – atrium, CD – copulatory duct, CO – copulatory opening, ET – epigynal teeth, FD – fertilization duct, SH – spermathecal head, Spe – spermatheca.

The female of this species resembles *T. variegata* (Wang, Yin, Peng & Xie, 1990) in having the slightly separated horn-like epigynal teeth located at the antero-median margin of a large atrium, and the short copulatory duct and spermathecae aggregated at the posterior part of vulvae, but can be separated from it by the large heart-shaped epigynal atrium (vs. horseshoe-shaped in *T. variegata*) and the copulatory ducts arising from sub-posteromedian part of the epigyne (vs. sub-posterolateral in *T. variegata*),and spermathecal heads pointing submedially towards the dorsal copulatory openings (Fig. 7C, D).

##### Description.

**Female. *Habitus*** as in Fig. 7A, B. Total length 7.32. Carapace 3.02 long, 1.79 wide. Eye sizes and interdistances: AME 0.13; ALE 0.17; PME 0.16; PLE 0.17; AME–AME 0.10; AME–ALE 0.06; PME–PME 0.10; ALE–ALE 0.39; PME–PLE 0.11; PLE–PLE 0.56; ALE–PLE 0.08; AME–PME 0.06; AME–PLE 0.25. MOA: 0.21 long; 0.26 anterior width, 0.38 posterior width. Chelicerae with large basal tubercle, three promarginal teeth (median largest) and two retromarginal teeth (proximal larger). Leg measurements: I 6.17 (1.48, 0.85, 1.55, 1.46, 0.83); II 5.63 (1.36, 0.79, 1.29, 1.38, 0.81); III 4.75 (0.96, 0.74, 0.98, 1.40, 0.67); IV 6.41 (1.19, 0.93, 1.56, 1.91, 0.82). Pedicel 2.54. Abdomen 3.68 long, 2.72 wide.

***Coloration*.** Carapace yellow-brown, posteriorly with dark radial stripes. Chelicerae, endites, labium, and sternum dark yellow-brown. Legs yellow-brown. Abdomen brown, dorsally with six pale chevron stripes on sub-medial part.

***Epigyne*** (Fig. 7C, D). Atrium, heart-shaped, rising from anterior to sub-posterior. Epigynal teeth thin and short, anteromedially located at the margin of atrium, separated by half length of epigynal teeth. Copulatory openings located at the posterior part of the atrium, separated by a narrow septum. Copulatory ducts ear-shaped, longer than epigynal teeth. Spermathecal heads finger-shaped, convergent, as long as half of epigynal teeth. Spermathecae sac-shaped, separated less than half of their lengths. Fertilization duct short, located posteriorly on spermathecae.

**Male.** Unknown.

##### Comments.

This species is characterized by the large atrium anteriorly located and the thin and short epigynal teeth anteromedially located. These characters are similar to those of *Tonsilla* species. Thus, this new species is tentatively placed in the genus *Tonsilla* until their matching males are found.

##### Distribution.

**Figure 8.**
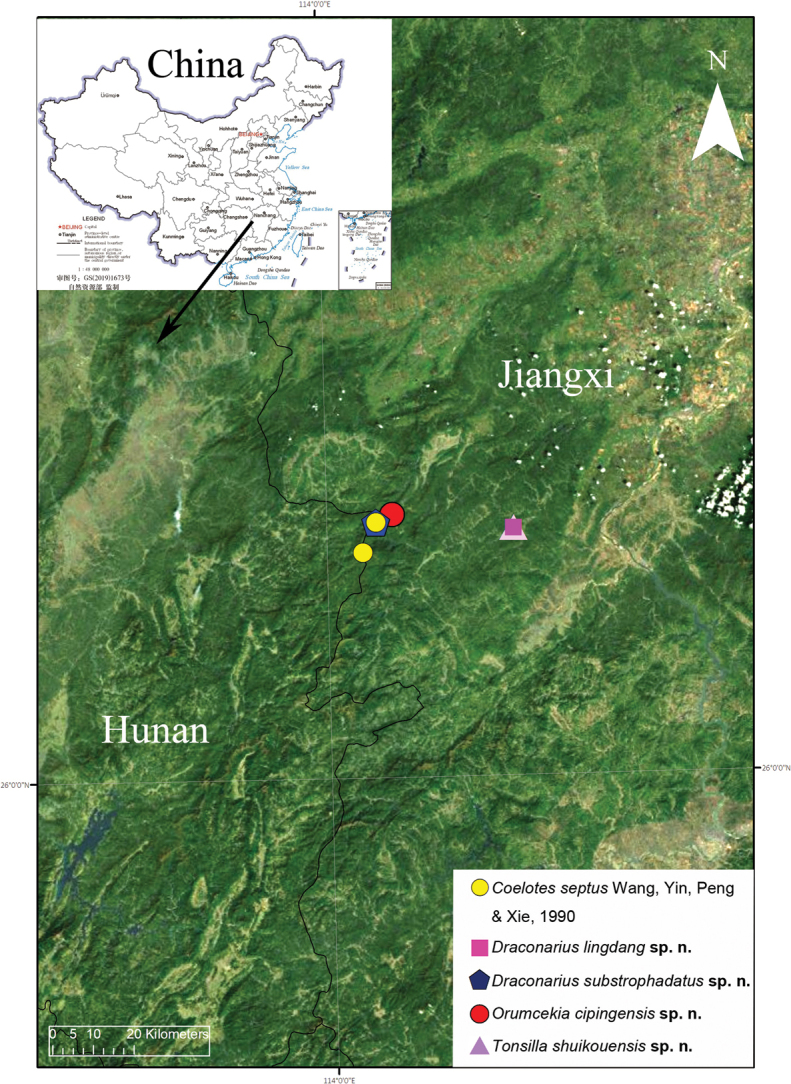
Type localities of *Coelotes septus*Wang et al., 1990, *Draconarius lingdang* sp. nov., *D. substrophadatus* sp. nov., *Orumcekia cipingensis* sp. nov., and *Tonsilla shuikouensis* sp. nov.

Known only from the type locality in Jiangxi Province, China (Fig. 8).

## Discussion

Coelotinae F.O. Pickard-Cambridge, 1893 is the largest subfamily among the spider family Agelenidae since it was transferred from Amaurobiidae by Miller et al. (2010) based on a phylogenetic analysis. The total number of species in Coelotinae has increased greatly in the last ten years (WSC, 2020). However, there are still many taxonomical problems to be resolved, especially in the two largest groups of genera, close to *Coelotes* and *Draconarius*. The main reason includes the following factors: firstly, ca. 70% species in these two genera are described from a single sex only; secondly, male species are very difficult to collect when in the mature period, because of the wandering behavior in this period searching for females; finally, some descriptions were superficial and only a few ink drawings were provided in previous work, causing difficulties in later species diagnosis. There is no doubt that the taxonomical work of these species has a long way to go.

It is interesting to note that some Chinese species, including those species described here, clearly appear to differ from some of their congeners, such as *C. septus*, which is characterized by the conspicuous tooth-like sclerite near the embolic base.

*Draconarius lingdang* shares common features with *D. aspinatus*, *D. peregrinus*, *D. potanini*, *D. rufulus*, *D. subabsentis*, and *D. tiantangensis* (Wang et al. 1990; Xu and Li 2008; Xie and Chen 2011). The main similarities in males are the absence of patellar apophyses, which exist in other *Draconarius* species. Unfortunately, the males of *D. substrophadatus*, *Orumcekia cipingensis*, and *Tonsilla shuikouensis* are still unknown. These problems need to be solved not only by the discovery of the unknown sexes, but also by analyzing the relationships using alternative methods, such as molecular studies.

## Supplementary Material

XML Treatment for Coelotes septus

XML Treatment for Draconarius lingdang

XML Treatment for Draconarius substrophadatus

XML Treatment for Orumcekia cipingensis

XML Treatment for Tonsilla shuikouensis
